# Probability Fluxes and Transition Paths in a Markovian Model Describing Complex Subunit Cooperativity in HCN2 Channels

**DOI:** 10.1371/journal.pcbi.1002721

**Published:** 2012-10-18

**Authors:** Klaus Benndorf, Jana Kusch, Eckhard Schulz

**Affiliations:** 1 Friedrich-Schiller-Universität Jena, Universitätsklinikum Jena, Institut für Physiologie II, Jena, Germany; 2 Fachhochschule Schmalkalden, Fakultät Elektrotechnik, Blechhammer, Schmalkalden, Germany; University of Notre Dame, United States of America

## Abstract

Hyperpolarization-activated cyclic nucleotide-modulated (HCN) channels are voltage-gated tetrameric cation channels that generate electrical rhythmicity in neurons and cardiomyocytes. Activation can be enhanced by the binding of adenosine-3′,5′-cyclic monophosphate (cAMP) to an intracellular cyclic nucleotide binding domain. Based on previously determined rate constants for a complex Markovian model describing the gating of homotetrameric HCN2 channels, we analyzed probability fluxes within this model, including unidirectional probability fluxes and the probability flux along transition paths. The time-dependent probability fluxes quantify the contributions of all 13 transitions of the model to channel activation. The binding of the first, third and fourth ligand evoked robust channel opening whereas the binding of the second ligand obstructed channel opening similar to the empty channel. Analysis of the net probability fluxes in terms of the transition path theory revealed pronounced hysteresis for channel activation and deactivation. These results provide quantitative insight into the complex interaction of the four structurally equal subunits, leading to non-equality in their function.

## Author Summary

The activation of a receptor protein by a small molecule (ligand) can be quantified by Markovian models. These models, which are widely used in natural sciences, consist of distinct states and transitions between them. In nature receptor proteins are often formed by the assembly of more than one subunit and each subunit can bind a ligand on its own. In such a multimeric receptor protein the translation of the ligand binding into receptor activation is more complex because the subunits interact. This usually limits the application of Markovian models. HCN2 pacemaker channels are tetrameric ion channels that mediate electrical rhythmicity in multiple brain and peripheral neurons and in specialized heart cells. The channels are modulated by cAMP binding to each subunit. We were recently successful to quantify ligand-induced activation for these channels by a complex Markovian model and a full set of rate constants. Herein we applied the transition path theory to further analyze the identified Markovian model, and we quantified time-dependent probability fluxes within the model. Our results provide unprecedented insight into the complex interaction of the four structurally equal subunits of a presumably fourfold symmetric channel that leads to pronounced non-equality of the subunit function.

## Introduction

In a protein the time scale of conformational changes ranges from picoseconds for the thermal vibration of the atoms to seconds, or even more, for structural arrangements [1]. Since proteins consist typically of hundreds to several thousand amino acids and each amino acid consists of seven or more atoms, the complete space of energetically possible states is huge, and certainly too huge for the experimentalist to study. It turned out, however, that often a set of a great many of these states can be viewed as metastable. This means that a protein typically fluctuates within a set of multiple different structures for a very long time if compared to the picosecond time scale of atom vibrations. Only extremely rarely the thermal energy suffices to leave a metastable set of states to reach another metastable set of states. A striking proof for this concept has been the discovery of the digital behavior of ion channels: Their pore is either closed or open, with dwell times often in the millisecond or even second time range [2]–[4].

Over the past decade there has been considerable progress in studying protein function theoretically by molecular dynamics (MD) simulations [5]. However, the maximum time range of presently possible MD simulations is still too short to become useful for the description of ion channel function. Kinetic analyses of ion channel gating are therefore presently conducted in terms of Markovian models, thereby treating a metastable set of states as one state and specifying transitions between two of these states according to Eyring's transition state theory [6]. Often, however, the accuracy of the experimental data does not suffice to differentiate between plausible models of sufficient complexity, e.g. to describe the action of three or more subunits. As a consequence the models used are often implausibly small or many simplifying assumptions for the equilibrium or rate constants have to be adopted.

Markovian models frequently used to describe the action of ion channels are either of the sequential or the cyclic type. Among the cyclic models the Monod-Wyman-Changeux (MWC) model [7] is of outstanding relevance because of its simple elegance: It assumes that a protein exists in two global conformations, taut and relaxed, and that each binding step systematically shifts the taut-relaxed isomerization and increases the binding affinity. This model, which has been most widely used to describe the sigmoidal oxygen-binding curve to hemoglobin (for review see [8]), has also been applied to describe the gating of ion channels by ligands, as e.g. the nicotinic acetylcholine receptor [9], cyclic nucleotide-gated (CNG) channels [10] or hyperpolarization activated cyclic nucleotide-modulated (HCN) channels [11]. However, as elegant as this model is, it does neither describe all aspects of the oxygen binding to hemoglobin [12], [13] nor is there any evidence that it fully describes the action of any ion channel [14]. For nicotinic acetylcholine and glycine receptors, the analysis of single-channel recordings has allowed the investigators to determine models with substantially more free parameters than typically employed by the MWC model. As a consequence, also the type of cooperativity differed substantially [15]–[18]. However, in all these approaches the ligand binding affinity could only be determined indirectly by the global fit of the channel activity, i.e. by the pore action, but not by direct measurement.

**Figure 1:**
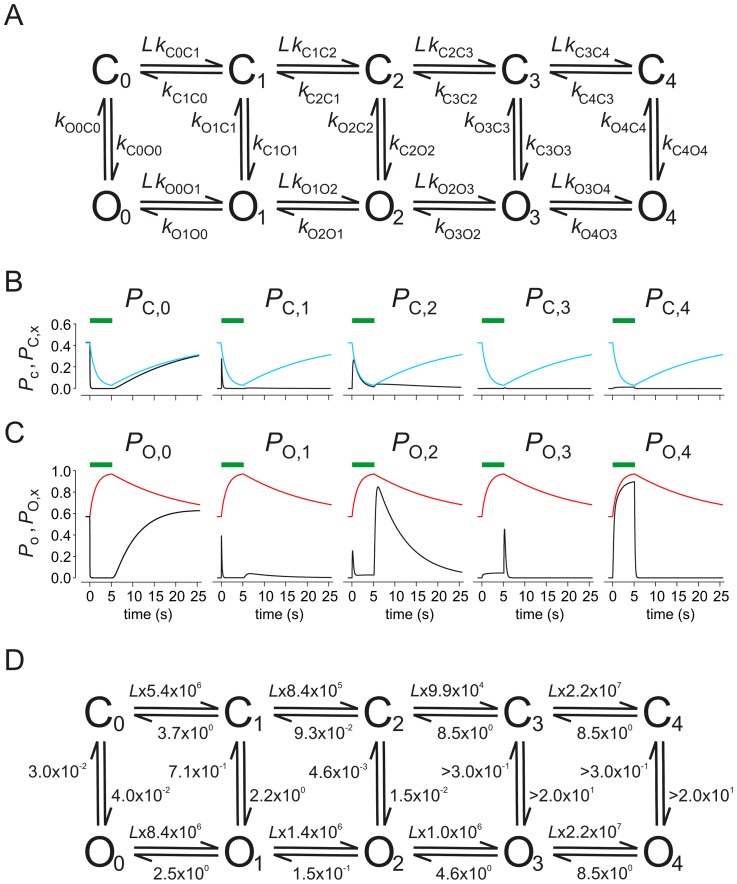
The C4L-O4L model. (*A*) The C4L-O4L model is composed of five closed (*C*_x_) and five open states (*O*_x_; x = 0…4). Ligand (*L*) binding is possible in both the closed and the open channel. The closed-open isomerization can proceed at each degree of liganding. The rate constants and their errors were determined previously [22]. *k*_O3C3_ was set equal to *k*_O4C4_. Since for these rate constants only a lower border could be estimated, they were set to 3 s^−1^ herein if not otherwise noted. This results in *k*_C3O3_ = *k*_C4O4_ = 2.0×10^2^ s^.1^. (*B*) Time-dependent probability to stay in a closed state, *P*_C,x_; (x = 0…4) for a ligand jump (green bar) from zero to 7.5 µM and back to zero. *P*_c_ denotes the sum of all *P*_C,x_ at each time. (*C*) Time-dependent probability to stay in an open state, *P*_O,x_; (x = 0…4) for a ligand jump (green bar) from zero to 7.5 µM and back to zero. *P*_o_, the sum of all *P*_O,x_ at each time, indicates the measured time course of the total open probability. (*D*) Scheme of the C4L-O4L model with means of rate constants provided by Table S1.

For CNGA2 and HCN2 channels we recently developed a strategy to simultaneously measure ligand binding and activation gating by combining confocal microscopy with patch-clamp fluorometry [19], thereby employing a fluorescently labeled ligand [20], [21]. In a subsequent study on HCN2 channels, pre-activated by a voltage pulse to −130 mV, we combined this approach with the method of concentration jumps. By globally fitting multiple time courses of ligand binding and channel activation, this approach allowed us to determine the equilibrium and rate constants in a Markovian model with 4 binding steps in both the closed and the open channel and 5 closed-open isomerizations (Fig. 1*A*) [22]. The analysis revealed pronounced cooperativity with respect to the microscopic binding affinity in the surprising sequence ‘positive – negative – positive’ for the binding of the second, third, and fourth ligand, respectively. Moreover, we considered the population of all closed and open states as function of time when jumping the ligand concentration. As a result, the total open probability is dominated by open states with either zero, two or four ligands bound whereas states with one or three ligands bound are only transiently populated (Fig. 1*B* and *C*) [22].

Herein we analyze the transition pathways in the C4L-O4L model [23], [24] when disturbing an equilibrium by a sudden change of a parameter. In our case this sudden change is either the application or the removal of a saturating ligand concentration. As a result the probability fluxes as function of time and the net probability fluxes are quantified, thereby identifying relevant and irrelevant transition pathways in channel activation and deactivation.

## Results

### Net probability flux densities in the C4L-O4L model

To specify the probability flux for any transition, we first consider a simple model with the states *A* and *B* and the two rate constants, *k*_1_ and *k*_−1_, specifying the transition rates.
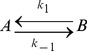
(Scheme 1)
*A* and *B* can also be interpreted as probabilities (with *A*+*B* = 1) which change under non-equilibrium conditions with time. At any time, a net probability flux density, *f*, can be defined by the sum of two opposed unidirectional fluxes. For example, the net probability flux density from *A* to *B* is given by *f*_AB_ = *k*_1_ *A*−*k*_−1_ *B*. If applying this to the individual transitions in the C4L-O4L model (for rate constants see Fig. 1*D*, Table S1), the net probability flux density of all transitions can be calculated at each time. In case of the binding reactions the rate constants have to be multiplied by the actual ligand concentration (either 0 or 7.5 µM). The arithmetic signs were chosen such that a probability flux is positive for the binding and opening reactions and, accordingly, negative for the unbinding and closing reactions. Because the probability of the states is time-dependent, the net probability flux density of each transition is also time-dependent.

**Figure 2:**
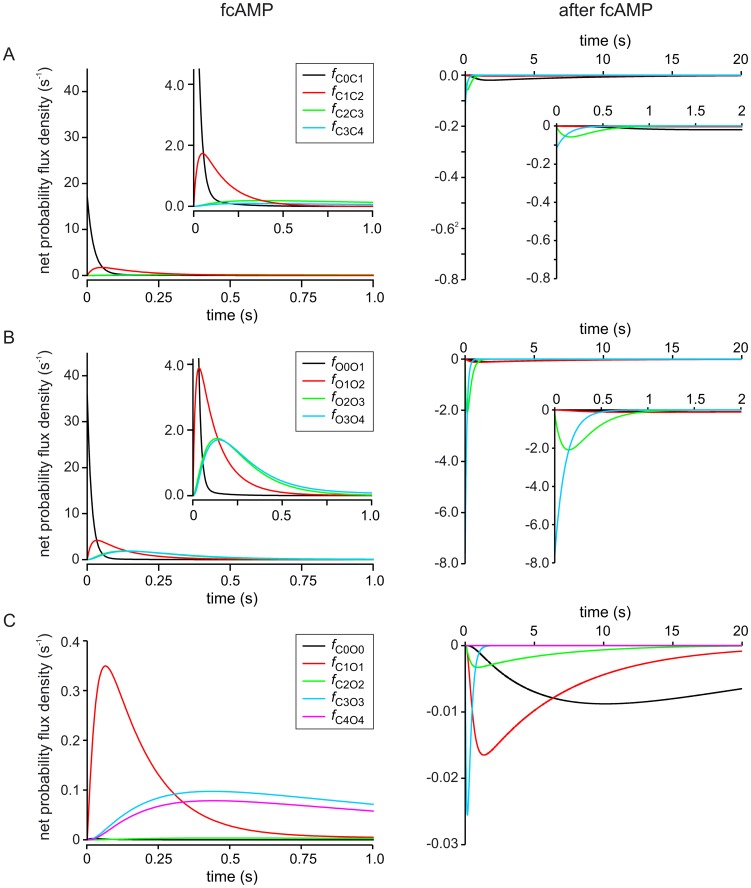
Time courses of net probability flux density in the C4L-O4L model. The time courses for the net probability flux density, *f*_XY_, in the C4L-O4L model were computed for the event of applying the ligand fcAMP (7.5 µM; left diagrams) and removing it again (right diagrams). The inset diagrams show the original diagram with an either higher amplitude or time resolution. (*A*) Net probability flux densities between closed states following ligand application (left) and removal (right) obtained by equation (1). (*B*) Net probability flux densities between open states following ligand application (left) and removal (right) obtained by equation (2). (*C*) Net probability flux density of the closed-open isomerizations following ligand application (left) and removal (right) obtained by equation (3).

First, the net probability flux density is considered when stepping from zero to 7.5 µM fcAMP. For the four binding steps in the closed channel, the net probability flux density is given by
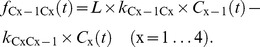
(1)
*L* is the ligand concentration. As expected, the net probability flux density moves like a wave from *C*_0_ to *C*_4_ (Fig. 2*A*, left): *f*_C0C1_ ceases after less than 100 ms and *f*_C1C2_ after about 500 ms. *f*_C2C3_ and *f*_C3C4_ are slower and not finished after 1s yet. The net probability flux density from the closed states to the respective open states contributes to the reduction of the net probability flux density between the closed states.

Accordingly, the net probability flux density for the four binding steps of the open channel, that has been pre-activated by voltage, is given by
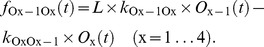
(2)


The respective time courses of *f*_Ox−1Ox_ are basically similar to those of the closed states but larger in amplitude (Fig. 2*B*, left). For the closed-open isomerizations the net probability flux density is given by
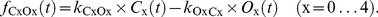
(3)


These time courses have a robust amplitude for *f*_C1O1_, *f*_C3O3_, and *f*_C4O4_ and are negligible for *f*_C0O0_ and *f*_C2O2_ (Fig. 2*C*, left). The obstruction of the pathway *C*_2_→*O*_2_ tells that the double liganded channel has a similarly taut structure as the non-liganded channel. The reduced degree of determinateness for *f*_C3O3_, and *f*_C4O4_ will be considered below.

Second, the net probability flux density of stepping from 7.5 µM fcAMP back to zero is considered. The respective time courses for the unbinding steps in the closed and open channel are given by equation (1) and (2), respectively, by setting *L* = 0 (Fig. 2*A and B*, right). The time courses for the open-closed isomerizations are given by equation (3) (Fig. 2*C*, right). The result is that the net probability flux density is much bigger in the unbinding steps of the open than of the closed channel and that *f*_O3O4_ and *f*_O2O3_ are much faster than *f*_O1O2_ and *f*_O0O1_, i.e. the state *O*_2_ is a severe obstacle in the net probability flux on the way to lower liganded states. Concerning the open-closed isomerizations, *f*_O4C4_ is negligible and *f*_O3C3_ is rapidly finished after about 1 s. In contrast, the other net probability fluxes are much slower. However, they all notably contribute to the closing process despite the rate constants for closing, *k*_O2C2_, *k*_O1C1_, and *k*_O1C1_, differ substantially. This shows how important it is to consider the model as a whole, including the rate constants *and* the time-dependent population of the states.

### Total net probability fluxes and transition paths in the C4L-O4L model

**Figure 3:**
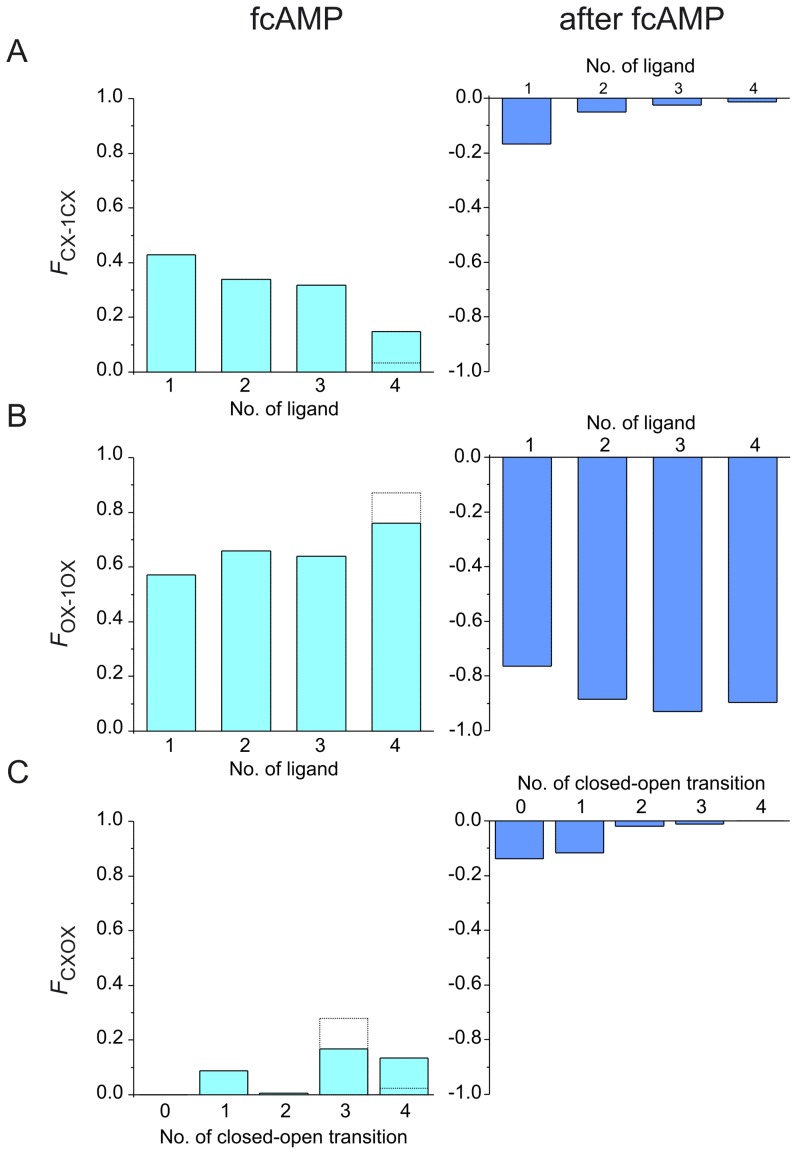
Total net probability fluxes in the individual transitions of the C4L-O4L model. The total net probability fluxes per transition, *F*_XY_ (given as fraction of unity), were obtained by integrating (equation (4)) the time courses of the net probability flux densities obtained by equation (1) to (3). The data after stepping to 7.5 µM fcAMP and after its removal are shown on the left and right side, respectively. The dotted bars indicate the effect of fitting with *k*_O3C3_ to 30, instead of 3, on *F*_C3C4_, *F*_O3O4_, *F*_C3O3_, and *F*_C4C4_. The sum of *F*_C3O3_ and *F*_C4C4_ is practically unchanged. The other total net probability fluxes were unchanged. (*A*) Total net probability fluxes for the transitions between neighbored closed states. (*B*) Total net probability fluxes for the transitions between neighbored open states. (*C*) Total net probability fluxes for the five closed-open transitions.

From the net probability flux densities, *f*_XY_, one can easily compute the total net probability fluxes, *F*_XY_, as the time integral over the time interval from the concentration jump (*t* = 0 s) to an end time, *t*_end_,
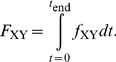
(4)
*t*_end_ is either 5 s for the fcAMP pulse or 20 s after removal of fcAMP. Adapted to our C4L-O4L model, *F*_XY_ indicates how much of the total net probability flux moves along a transition *X*↔*Y*. The main results are: (1) The total net probability flux is bigger in the binding steps of the open than the closed channel in both the presence of fcAMP and after its removal (Fig. 3*A and B*). (2) The total net probability flux between the closed states is larger for the binding than for the unbinding process (Fig. 3*A*). (3) *F*_C0O0_ is negligible in the binding-induced relaxation but significantly present in the unbinding-induced relaxation whereas *F*_C4O4_ is negligible in the unbinding-induced relaxation but significantly present in the binding-induced relaxation (Fig. 3*C*). Together, these results suggest different pathways for activation and deactivation.

To gain a more thorough insight into the transition pathways in our C4L-O4L model, they were analyzed according to the principles of the transition path theory [23], [25]. In this type of analysis one is interested in trajectories between two selected states within the model. This implies that all repeated jumps between two neighbored states in both directions are not counted; i.e. considered are only transitions in one direction. This information is hidden in the total net probability flux for the individual transitions, *F*_XY_, which we determined in the previous section. In any model the amount of net flux produced by one set of states must equal the amount of net flux collected by the other set of states. When applying the ligand, the only flux producers are the states *C*_0_ and *O*_0_. Because the applied ligand concentration was saturating, the main flux collector state is *O*_4_ while *O*_3_ and *O*_2_ also contribute but to an only small extent (Fig. 1*C*). After removing the ligand, the main flux producer state is *O*_4_, and to a small extent *O*_3_ and *O*_2_. The main flux collector states are *C*_0_ and *O*_0_. The states *C*_2_ and *O*_2_ are, to a minor extent, also flux collector states because after 20 s the steady state was not reached (Fig. 1*B* and *C*).

In case of a fully reversible Markovian model, which holds for the C4L-O4L model, the total probability flux can easily be decomposed into the pathway net probability fluxes, and no cycles remain because the condition of the detailed balance is fulfilled [22]. The procedure is to choose first a pathway from state *X*_0_ to state *X*_k_ along the states *X*_i_. Then for this chosen pathway, *X*_0_→*X*_k_, the net probability flux *F*_p,X0Xk_ is computed according to
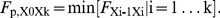
(5)
*F*_p,X0X_ is then removed from the flux along all edges of this pathway and the procedure is repeated for the remaining pathway net probability fluxes until the total possible probability flux between these states is zero [25]. It should be noted that the sequence of such a decomposition is not unique because different paths can be chosen. It has become useful to start with the strongest pathway [25].

**Figure 4:**
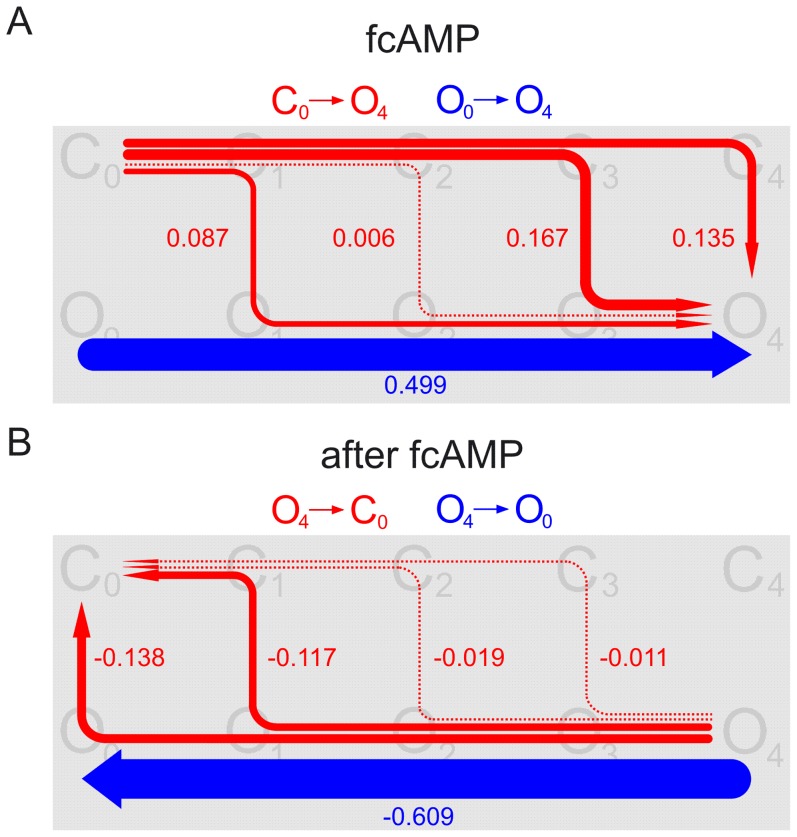
Transition pathways in the C4L-O4L model at saturating fcAMP. The weight of the pathway net probability fluxes, obtained by equation (5), is given by the numbers besides the colored graphs (as fraction of unity) and, for values >0.030, also encoded by the thickness of the arrows. Fluxes from 0.002 to 0.030 are illustrated by equally thick dotted arrows. All other fluxes are only very small (<4.5×10^−4^) and therefore not represented by arrows. For further explanation see text. (*A*) Pathway net probability fluxes along the pathways *C*_0_ to *O*_4_ (red) and *O*_0_ to *O*_4_ (blue) after switching to 7.5 µM fcAMP. (*B*) Pathway net probability fluxes along the pathways *O*_4_ to *C*_0_ (red) and *O*_4_ to *O*_0_ (blue) after removing fcAMP.

Let us first consider the pathway net probability flux after applying the ligand at 7.5 µM fcAMP (Fig. 4*A*). The pathway net probability flux from *C*_0_, one of the two flux producers, to *O*_4_, the main flux collector, results in five defined pathway fluxes, *F*_p,_, which are, according to equation (5), given by the respective minimum values of *F*_CxOx_ (x = 0…4). The pathway net probability flux from *O*_0_, the other flux producer, to *O*_4_ has only one available path because the fluxes of all closed-open transitions are directed to the respective open states. Illustration of the weights of the pathway net probability fluxes in the C4L-O4L model after applying the ligand leads to the result that ligand-induced channel opening proceeds mainly from *C*_3_ and *C*_4_ (with the uncertainty of the exact attribution; Fig. 3, left), and additionally from *C*_1_, but not relevantly from *C*_0_ and *C*_2_. The same type of analysis was then performed for the pathway net probability flux after removing the ligand from *O*_4_, the main flux producer, to *C*_0_ and *O*_0_, the main flux collectors. Closing of the channels proceeds predominantly from *O*_1_ and *O*_0_, and to a minor extent from *O*_2_ and *O*_3_, but not relevantly from *O*_4_. Together these results show that there is pronounced hysteresis for ligand-induced activation and deactivation. The pathway flux from *O*_4_ to *O*_0_, the other flux collector, has again only one available path because the fluxes of all closed-open transitions are directed to the respective closed states.

**Figure 5:**
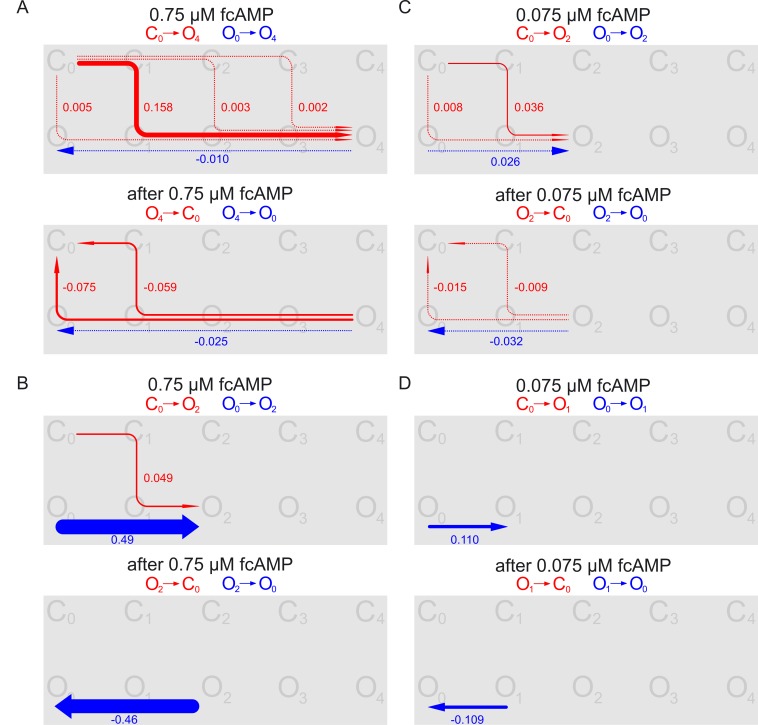
Transition pathways in the C4L-O4L model at subsaturating fcAMP. The illustration is analogous to Fig. 4. The two main net probability fluxes at each concentration are shown. Pathway net probability fluxes associated with activation (*C*_0_→*O*_x_, *O*_0_→*O*_x_) and deactivation (*C*_x_→*O*_0_, *O*_x_→*O*_0_) are shown in red and blue color, respectively. (*A*) 0.75 µM fcAMP, *C*_0_↔*O*_4_. (*B*) 0.75 µM fcAMP, net probability flux along *C*_0_↔*O*_2_ in addition to that included in *C*_0_↔*O*_4_. (*C*) 0.075 µM fcAMP, *C*_0_↔*O*_2_. (*D*) 0.075 µM fcAMP, net probability flux along *C*_0_↔*O*_1_ in addition to that included in *C*_0_↔*O*_2_.

To demonstrate the function of our model at subsaturating fcAMP concentrations we considered the fluxes to the two main collectors at 0.75 µM fcAMP (*O*_2_ and *O*_4_; Fig. 5 *A*,*B*) and 0.075 µM fcAMP (*O*_1_ and *O*_2_; Fig. 5 *C*,*D*) and the respective reverse fluxes when removing fcAMP. Notably, these selected fluxes are only the dominating fluxes. The results show that at the intermediate concentration of 0.75 µM fcAMP activation proceeds along *C*_1_→*O*_1_ predominantly to *O*_4_ (Fig. 5 *A*) and to a minor extent to *O*_2_ (Fig. 5 *B*). In addition there is a remarkably big net probability flux in the open channel along *O*_0_→*O*_2_ and in the reverse direction along *O*_2_→*O*_0_ which is absent along *O*_0_→*O*_4_ and *O*_4_→*O*_0_, respectively. This indicates that *O*_2_ is a metastable state. Moreover, this result corresponds to the high energy barrier for the transition *O*_2_→*O*_3_ [22]. At 0.075 µM fcAMP the predominant activation proceeds along *C*_1_→*O*_1_ to *O*_2_ but not anymore to *O*_4_. *O*_1_ is only passed in the activation pathway. However, it is of importance as a collector for the probability flux in the open channel (Fig. 5 *D*).

### Unidirectional fluxes in the closed-open isomerization as function of time

In our considerations on probability fluxes so far the focus was set on net probability fluxes. If a flux is reversible, as in case of ligand binding and closed-open isomerizations, a net flux consists of two opposed unidirectional fluxes. Generally, very different opposed unidirectional fluxes can cause the same net flux. Large opposed unidirectional fluxes indicate conformational flexibility between two states, whereas small opposed unidirectional fluxes indicate conformational tautness between two states.

**Figure 6:**
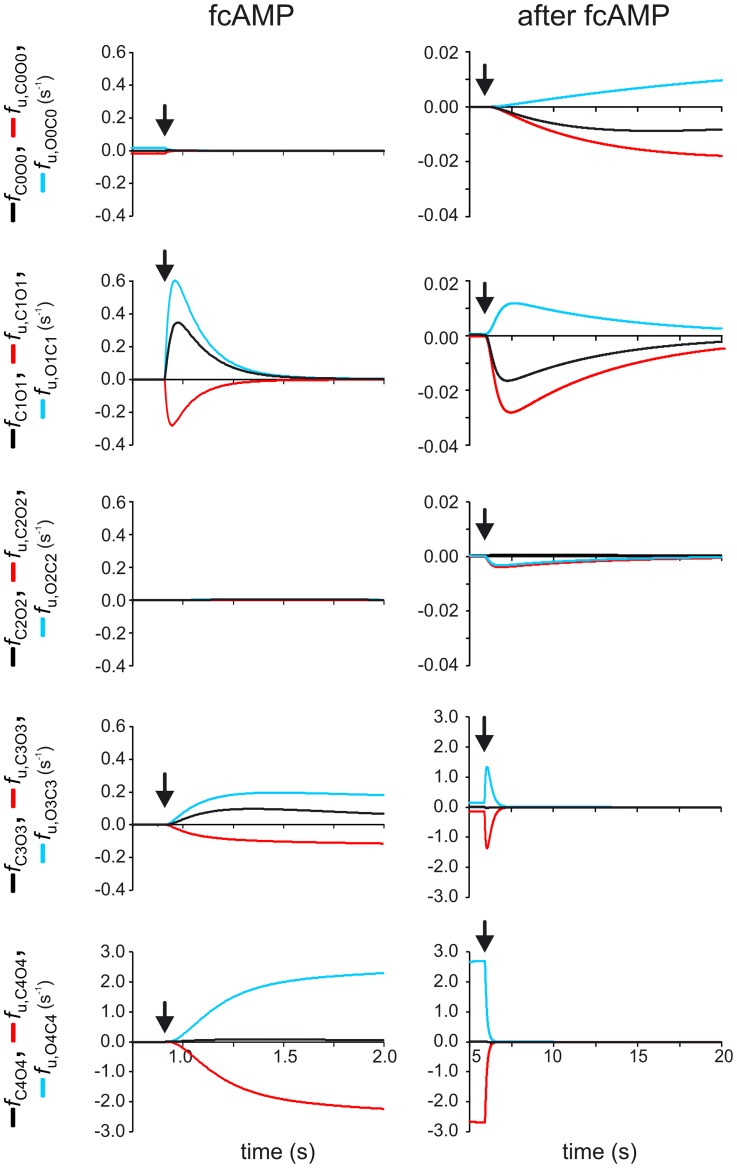
Unidirectional probability flux densities in the closed-open isomerizations. The time courses of the unidirectional probability flux densities, *f*_U,CxOx_ and *f*_U,OxCx_ (x = 0…4) for the closed-open isomerizations are plotted together with the respective net probability flux densities, *f*_CxOx_, when applying 7.5 µM fcAMP (left) and removing it (right). The arrows indicate the time point of concentration change. For explanation see text.

For the principal conformational change of the closed-open isomerization we considered the time courses of the unidirectional probability flux densities, *f*_U,CxOx_>0 and *f*_U,OxCx_<0 (x = 0…4), at the saturating fcAMP concentration of 7.5 µM fcAMP and related them to the net probability flux density, *f*_CxOx_ (Fig. 6; c.f. Fig. 2*C*), according to
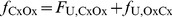
(6)For the time after application of fcAMP, these time courses show that the transitions *C*_0_↔*O*_0_ and *C*_2_↔*O*_2_ are functionally irrelevant. In contrast, the net probability flux densities in the transitions *C*_1_↔*O*_1_ and *C*_3_↔*O*_3_ are robust and the unidirectional probability flux densities exceed the respective net probability flux density substantially. An extreme surplus of the unidirectional probability flux densities with respect to the net probability flux density is inherent in the transition *C*_4_↔*O*_4_, suggesting pronounced conformational flexibility when the channel is fully liganded. It is also notable that the empty activated channel (Fig. 6, top left) is much less flexible in this sense compared to the fully liganded channel (Fig. 6, bottom left).

After removal of fcAMP (Fig. 6, right), the fully liganded state is left rapidly (bottom) and the triple liganded state is rapidly passed. For the remaining transitions *C*_2_↔*O*_2_, *C*_1_↔*O*_1_, and *C*_0_↔*O*_0_ both the maximum net and the maximum unidirectional probability flux densities are much smaller than for *C*_4_↔*O*_4_ and *C*_3_↔*O*_3_. The finally large total net probability flux in the transitions *C*_2_↔*O*_2_, *C*_1_↔*O*_1_, and *C*_0_↔*O*_0_ (c.f. Fig. 3*C*, right) is only reached after many seconds. Remarkably, in the transition *C*_2_↔*O*_2_ the unidirectional probability flux density in the opening direction approximates zero, which is due to the low occupancy of *C*_2_, resulting in a close similarity of the unidirectional closing and the net probability flux density. This contrasts to *C*_1_↔*O*_1_ and *C*_0_↔*O*_0_ which both show larger unidirectional probability flux densities than the respective net probability flux density. Both the net and the unidirectional flux densities in the transition *C*_0_↔*O*_0_ reach finally the respective initial values before applying fcAMP (Fig. 6*A*, top left). The results in Fig. 6 also suggest that the binding of two ligands reduces the conformational flexibility of the closed-open isomerization maximally and, more generally, how different the effects of the four binding steps on the closed-open isomerization are.

## Discussion

### The C4L-O4L model

We present a detailed analysis of probability fluxes within a Markovian model describing ligand-induced activation of HCN2 channels. The analysis is based on the combined recording of ligand binding and channel activation [22]. The main results are that significant activation of the channel proceeds with one, three, and four ligands bound whereas significant deactivation proceeds relevantly with two, one and zero ligands bound. The consequence of this result is a pronounced hysteresis for channel activation and deactivation. In addition to this we show that the channel is in a flexible conformation with one, three, and four ligands bound whereas it is in a taught conformation with zero and two ligands bound. Notably, we herein considered at each degree of liganding probability fluxes for the whole channel, i.e. we did not use specific stoichiometric factors. This allowed us to avoid any further assumptions concerning equivalence or non-equivalence of the available binding sites.

The high degree of determinateness of our approach was certainly not only caused by analyzing data of ligand binding and gating simultaneously, but also by the specific nature of the effect induced by the concentration jumps: Applying the ligand abruptly transformed the simple *C*_0_↔*O*_0_ model into the complex C4L-O4L model. Conversely, removing the ligand emptied the C4L-O4L model and led to the initial *C*_0_↔*O*_0_ model. Only few parameters were not determined. The limited time resolution of our method did not allow us to distinguish some of the rates with three and four ligands bound. It should be emphasized, however, that none of the present conclusions depends on specific assumptions in the fit.

### Transition path analysis in ion channel gating

For analyzing the action of proteins great progress has been achieved over the past years by molecular dynamics (MD) simulations and building energy landscapes. In these landscapes often a small set of energy basins can be identified that are separated by energy barriers (for review see [26]). However, present MD simulations are typically limited by the available computer technologies to one microsecond [5] or, very recently, even to a millisecond [27]–[29], thereby already touching the time range of the observables in ion channel gating. In contrast, functional measurements are real and they provide a different kind of information about the action of ion channels compared to MD simulations: The pore opening can be monitored by the ion current, distinct conformational changes by changes in the fluorescence intensity of introduced labels [30]–[32] and, in case of voltage-gated channels, the movement of the gating apparatus by gating currents [33]–[35]. Markovian state models are often used to interpret functional data.

Over the past decade there has been great progress in applying the transition path theory in combination with Markovian state models to learn more about the action of proteins. However, this was done only in theory by MD simulations and in the mentioned short time range [23], [25], [36]. Also graphical visualization of the paths has been performed [37]. So far, the transition path theory has not been applied to the gating of ion channels as performed herein.

Our approach quantifies at which degree of liganding the closed-open isomerization proceeds and demonstrates a complex type of hysteresis in channel gating (Fig. 4). Though this information is, of course, determined by the rate constants, the complexity of the C4L-O4L model precludes an immediate identification. Such a hysteresis might have physiological consequences for the regulation of the channels. For example, molecules regulating the activity of these channels might have different affinities at different conformations. Also, knowledge of the transition pathways might become very helpful for future strategies to develop drugs stimulating or blocking these channels. For example, if one intends to reduce the cAMP effect on the open probability of HCN2 channels in a living cell, it might be a good idea to selectively affect the *C*_1_↔*O*_1_ transition because it is this transition which mediates the main effect in the lower concentration range.

### Net and unidirectional probability flux densities

In addition to the sum of the probability fluxes determined by the transition path analysis, our approach provided us dynamic information: the net and unidirectional probability flux densities. The net probability flux density shows the flux for the different transitions as function of time, i.e. how often per time interval a net transition appears (Fig. 2). There are two important aspects of this information: First, these time courses show the maximum intensities of the transitions. Second, these time courses show how differently rapid these transitions are. This might be also of interest for the development of highly specific drugs modulating the channel activities. Moreover, this knowledge might be of relevance for learning how the subunits interact, how the identified complex cooperativity [22] is generated by subunits that are equal in their amino acid sequence.

The unidirectional probability flux density (Fig. 6) provides another type of information: It specifies all transitions from one state to a neighbor state, including also the repetitive transitions. The unidirectional probability flux density can be much bigger than the net probability flux density. Hence the unidirectional probability flux density provides information how taut or relaxed an HCN2 channel is at a given degree of liganding. The most challenging result is that the tautness is not a monotonous function of the degree of liganding, it is high when none or two ligands are bound and low when one, three and four ligands are bound (Fig. 6, left). In analogy to the T and R conformation in hemoglobin [8], this result suggests that the binding of the first, third and fourth ligand leads to a break of hydrogen bonds whereas the binding of the second ligand promotes the formation of hydrogen bonds.

One might be inclined to relate our results to entropy changes associated with the transition state upon channel activation under the assumption of reducing the complex activation to a single transition and employing Eyring's rate theory [6]. This has been performed for multiple channels, e.g. voltage-gated K^+^ channels [38]–[40] and voltage-gated Na^+^ channels [41]. For related CNGA2 channels we reported previously that the entropy of the open channel plus its environment is smaller than that of the closed channel plus its environment [42], similar to the results in the voltage-gated K^+^ and Na^+^ channels. For HCN channels respective data are missing.

The fact that the largest unidirectional probability flux density observed herein was observed for the fully liganded channel seems to suggest that the entropy of the channel is higher in the fully liganded state than in the incompletely liganded state. However, this measure of channel flexibility is fundamentally different from a thermodynamic entropy because the environment is not considered. Therefore, the elevated channel flexibility at full liganding indicates a selective property of closed-open isomerization, irrespective of its environment. For further analysis of the gating process in HCN2 channels it would be a good idea to repeat our experiments at different temperatures and study the temperature dependency of the individual rate constants.

### Conclusion

We analyzed the gating of homotetrameric HCN2 channels by probability fluxes in a complex Markovian model, the C4L-O4L model. This analysis was not based on simulations but on a fit to functional experimental data. Studying time courses of the net probability flux density, the unidirectional probability flux density, the total net probability flux, and the transition paths within the C4L-O4L model provided us an unusual view on the gating of the channels. Most remarkably, there is considerable ligand-induced channel opening already after the first ligand has bound whereas there is practically no further opening after the second ligand has bound. Moreover, our analysis shows pronounced hysteresis associated with channel opening and closure. Our results should help to better understand the physiological function of HCN channels and, possibly, to develop strategies for a pharmacological modulation of special functional states.

## Materials and Methods

All calculations are based on a kinetic model for the ligand-induced activation of HCN2 channels containing four ligand binding steps in both the closed and open channel and five closed-open isomerizations (C4L-O4L model termed herein; Fig. 1*A*) [22]. The channels were activated by a voltage pulse from −30 mV to −130 mV and fcAMP was applied only after the voltage-induced activation was maximal. Fixing the voltage to −130 mV provided us the advantage to study the ligand-induced gating independent of any intervening effects of a changed voltage. The rate constants, determined previously by a global fit strategy of multiple time courses of ligand binding and unbinding as well as activation and deactivation gating, are listed together with their s.e.m. in Table S1. The time courses in the present study were computed by using the mean rate constants. The time-dependent occupancies of the states were computed with the Eigenvalue method using Matlab®. Initially, the channel was assumed to be in the equilibrium *C*_0_↔*O*_0_ because the ligand concentration, *L*, was zero (Fig. 1). Then *L* was set to the value of the applied fcAMP concentration (activation) and back to zero (deactivation). For the numerical computation of time integrals, Simpson coefficients were used.

## Supporting Information

Table S1 **Rate constants for the C4L-O4L model.** The rate constants were computed by the global fit as described [22].
(DOC)
