# Supplementary material for: Probability Fluxes and Transition Paths in a Markovian Model Describing Complex Subunit Cooperativity in HCN2 Channels
Source: PLoS Comput Biol. 2012 Oct 18;8(10):e1002721. doi: 10.1371/journal.pcbi.1002721 (PMC3475657; doi:10.1371/journal.pcbi.1002721)
Supplement: Table S1 — Rate constants for the C4L-O4L model. The rate constants were computed by the global fit as described [22]. (DOC) [file pcbi.1002721.s001.doc]

Supporting Information

**Probability fluxes and transition paths in a Markovian model describing complex subunit cooperativity in HCN2 channels**

# Klaus Benndorf, Jana Kusch, Eckhard Schulz

Supporting Tables

**Table S1.** Rate constants for the C4L-O4L model.

| Parameter | Dimension | Value | s.e.m. | s.e.m. (%) |
| --- | --- | --- | --- | --- |
| *k*_C0C1_ | s^-1^M^-1^ | 5.4x10^6^ | 4.2x10^5^ | 7.78 |
| *k*_C1C0_ | s^-1^ | 3.7x10^0^ | 3.1x10^-1^ | 8.38 |
| *k*_C1C2_ | s^-1^M^-1^ | 8.4x10^5^ | 3.9x10^4^ | 4.64 |
| *k*_C2C1_ | s^-1^ | 9.3x10^-2^ | 4.3x10^-3^ | 4.62 |
| *k*_C2C3_ | s^-1^M^-1^ | 9.9x10^4^ | 1.6x10^3^ | 1.62 |
| *k*_C3C2_ | s^-1^ | 8.5x10^0^ | 5.2x10^-1^ | 6.12 |
| *k*_C3C4_ | s^-1^M^-1^ | 2.2x10^7^ | 3.2x10^6^ | 14.6 |
| *k*_C4C3_ | s^-1^ | 8.5x10^0^ | 5.2x10^-1^ | 6.12 |
|  |  |  |  |  |
| *k*_O0O1_ | s^-1^M^-1^ | 8.4x10^6^ | 5.4x10^5^ | 6.43 |
| *k*_O1O0_ | s^-1^ | 2.5x10^0^ | 1.7x10^-1^ | 6.80 |
| *k*_O1O2_ | s^-1^M^-1^ | 1.4x10^6^ | 3.6x10^4^ | 2.57 |
| *k*_O2O1_ | s^-1^ | 1.5x10^-1^ | 1.5x10^-3^ | 1.00 |
| *k*_O2O3_ | s^-1^M^-1^ | 1.0x10^6^ | 9.2x10^4^ | 9.20 |
| *k*_O3O2_ | s^-1^ | 4.6x10^0^ | 3.6x10^-1^ | 7.83 |
| *k*_O3O4_ | s^-1^M^-1^ | 2.2x10^7^ | 3.2x10^6^ | 14.6 |
| *k*_O4O3_ | s^-1^ | 8.5x10^0^ | 5.2x10^-1^ | 6.12 |
|  |  |  |  |  |
| *k*_O0C0_ | s^-1^ | 3.0x10^-2^ | 2.7 x10^-3^ | 9.00 |
| *k*_C0O0_ | s^-1^ | 4.0x10^-2^ | 3.6 x10^-3^ | 9.00 |
| *k*_O1C1_ | s^-1^ | 7.1x10^-1^ | 6.2 x10^-2^ | 8.73 |
| *k*_C1O1_ | s^-1^ | 2.2x10^0^ | 1.8 x10^-1^ | 8.18 |
| *k*_O2C2_ | s^-1^ | 4.6x10^-3^ | 1.6 x10^-3^ | 34.9 |
| *k*_C2O2_ | s^-1^ | 1.5x10^-2^ | 5.4 x10^-3^ | 36.0 |
| *k*_O3C3_ | s^-1^ | >3x10^-1^→ 3x10^0^ |  |  |
| *k*_C3O3_ | s^-1^ | >2x10^1^→ 2x10^2^ |  |  |
| *k*_O4C4_ | s^-1^ | >3x10^-1^→ 3x10^0^ |  |  |
| *K*_C4O4_ | s^-1^ | >2x10^1^→ 2x10^2^ |  |  |

The rate constants were computed by the global fit as described ]. *k*_O3C3_=*k*_O4C4_ was not determined by the fit, however, a lower border of 0.3 s^-1^ was determined by a 10% increase of *χ*^2^. For the computations herein we used ten times this value, 3 s^-1^, resulting in *k*_C3O3_=*k*_C4O4_=2.0×10^2^ s^-1^.
